# Can we measure individual differences in cognitive measures reliably via smartphones? A comparison of the flanker effect across device types and samples

**DOI:** 10.3758/s13428-022-01885-6

**Published:** 2022-06-16

**Authors:** Thomas Pronk, Rebecca J. Hirst, Reinout W. Wiers, Jaap M. J. Murre

**Affiliations:** 1 Department of Psychology, Faculty of Social and Behavioural Sciences, University of Amsterdam, P.O. Box: 15804, 1001 NH Amsterdam, The Netherlands; 2 Behavioural Science Lab, Faculty of Social and Behavioural Sciences, University of Amsterdam, Amsterdam, The Netherlands; 3 Open Science Tools (PsychoPy) Lab, School of Psychology, University of Nottingham, Nottingham, UK

**Keywords:** Flanker effect, Experimental effects, Individual differences, Reliability, Internet, Smartphones, Web applications

## Abstract

Research deployed via the internet and administered via smartphones could have access to more diverse samples than lab-based research. Diverse samples could have relatively high variation in their traits and so yield relatively reliable measurements of individual differences in these traits. Several cognitive tasks that originated from the experimental research tradition have been reported to yield relatively low reliabilities (Hedge et al., 2018) in samples with restricted variance (students). This issue could potentially be addressed by smartphone-mediated administration in diverse samples. We formulate several criteria to determine whether a cognitive task is suitable for individual differences research on commodity smartphones: no very brief or precise stimulus timing, relative response times (RTs), a maximum of two response options, and a small number of graphical stimuli. The flanker task meets these criteria. We compared the reliability of individual differences in the flanker effect across samples and devices in a preregistered study. We found no evidence that a more diverse sample yields higher reliabilities. We also found no evidence that commodity smartphones yield lower reliabilities than commodity laptops. Hence, diverse samples might not improve reliability above student samples, but smartphones may well measure individual differences with cognitive tasks reliably. Exploratively, we examined different reliability coefficients, split-half reliabilities, and the development of reliability estimates as a function of task length.

## Introduction

Psychology has been called a science of two research traditions: the experimental and correlational traditions (Cronbach, 1957). The experimental tradition focuses on identifying cognitive processes that are universal across individuals (Cronbach, 1957), while the correlational tradition focuses on the assessment of individual differences (e.g., IQ). The presence of a cognitive process has traditionally been demonstrated by detecting the presence of an experimental effect. This effect is produced by manipulating a cognitive process, which in turn affects performance on an outcome measure such as the correctness or speed of a response (Goodhew & Edwards, 2019). Variation between individuals in the magnitude of an experimental effect decreases the power with which it can be detected. Hence, a relatively homogeneous sample might reveal an experimental effect more easily than a diverse sample. In contrast, the correlational tradition focuses on identifying the relationship between cognitive processes across individuals. Identifying such relationships requires variation between participants, i.e., individual differences in the magnitude of experimental effects. Hence, a relatively diverse sample might reveal individual differences more easily than a homogeneous sample.

Cognitive tasks originating from the correlational tradition, such as simple reaction time, have been demonstrated to measure individual differences reliably (Baker et al., 1986; Hamsher & Benton, 1977). However, it has been disputed whether cognitive tasks originating from the experimental tradition can measure individual differences reliably. A landmark paper in this controversy presented a replication of seven classical cognitive tasks across three studies (Hedge et al., 2018). Estimated via test-retest and split-half methods, all replications but one found reliability coefficients below 0.8. The finding that many cognitive tasks are perfectly suitable for detecting experimental effects, but apparently unsuitable for measuring individual differences was coined by Hedge et al. (2018) as a “reliability paradox”. These replications were conducted in a lab setting in a relatively homogeneous sample, namely psychology undergraduates. Hence, one possible explanation for the low reliabilities could be a lack of diversity in the sample, and consequentially, a lack of sufficient variation in the magnitude of experimental effects between participants.

Diverse samples can be relatively difficult to recruit for research in lab settings, but perhaps easier via the internet (Birnbaum, 2004; Reips, 2000; Woods et al., 2015). Web applications are a suitable technology for administering cognitive tasks deployed via the internet because web applications are based on open standards (such as HTML, CSS, and JavaScript) and are supported by web browsers across a wide range of devices. This allows web applications to be administered not only on keyboard devices (such as desktops and laptops) but also on touchscreen devices (such as tablets and smartphones). Ownership of keyboard devices can be limited to a relatively affluent stratum of the population, but smartphone ownership is increasingly ubiquitous (O’Dea, 2022; Pew Research Center, 2016). Hence, research deployed via the internet and administered via smartphones may have access to diverse samples (Dufau et al., 2011), and so provide more reliable measurements of individual differences.

Establishing whether this potential can be fulfilled requires systematic empirical verification, for which the current study aims to be the first step. Below, we first review extant studies that replicated effects with cognitive tasks that were implemented as web applications. Secondly, we review the timing accuracy of web applications, followed by a review of the unique features of smartphone user interfaces. We distill some design guidelines for cognitive tasks administered as web applications on smartphones. Finally, we justify our selection of the flanker effect for the focus of this study; arguing why data collected via the Eriksen flanker task may show higher reliabilities in more diverse samples and why this task may be suitable for smartphone administration. Based on these reviews, we designed a study that systematically compares samples and device types.

### Replicating experimental effects with cognitive tasks as web applications

Studies that have administered cognitive tasks via web applications inside and outside of the lab have generally replicated experimental effects found with these tasks (Barnhoorn et al., 2015; Bazilinskyy & de Winter, 2018; Crump et al., 2013; de Leeuw & Motz, 2016; Frank et al., 2016; Germine et al., 2012; Hilbig, 2016; Semmelmann et al., 2016; Semmelmann & Weigelt, 2017). Whereas most tasks were administered on keyboard devices, some of these replications were administered on tablets (Frank et al., 2016; Semmelmann et al., 2016) and one on smartphones (Bazilinskyy & de Winter, 2018). Most studies were conducted on student samples, with some studies including non-student samples, such as convenience samples or samples recruited via Amazon Mechanical Turk (Bazilinskyy & de Winter, 2018; Crump et al., 2013; Germine et al., 2012; Semmelmann, 2017). One study examined the reliability of measuring individual differences with internal consistency methods such as Cronbach’s alpha and first-second split-half reliability, stratified by task conditions (Germine et al., 2012). Across four non-student samples and five cognitive tasks, Germine et al. (2012) found reliability estimates that were comparable to lab studies.

The studies above showed promising results in replicating experimental effects on keyboard devices. However, only a small number of studies administered cognitive tasks via smartphones or examined the reliability with which individual differences could be measured. None have performed a systematic comparison of samples (student versus non-student) and devices (laptops versus smartphones). We conducted such a systematic comparison, with a focus on individual differences instead of experimental effects.

### Timing accuracy of commodity devices

When studies are deployed via the internet, they are administered on commodity devices (i.e., devices owned by participants). Doubts have been voiced about whether these devices are sufficiently accurate at timing stimuli and measuring response times (RTs) (Plant & Quinlan, 2013; van Steenbergen & Bocanegra, 2016). Studies that have measured stimulus duration via photometry on desktops and laptops, found that accuracy varied per combination of physical device, operating system, and browser, henceforth jointly denoted as device (Anwyl-Irvine et al., 2021; Barnhoorn et al., 2015; Bridges et al., 2020; Garaizar et al., 2014; Garaizar & Reips, 2019; Reimers & Stewart, 2015). In a recent study that included smartphones, duration accuracy ranged from near-perfect in best-case scenarios, to worst-case scenarios where timing could be off up to 66.6 ms (Pronk et al., 2020). This renders cognitive tasks that require particularly short or precise stimulus durations less suitable for administration on commodity devices.

Measurements of RT may make noisy overestimations, with the mean and variance of this noise varying across devices (Anwyl-Irvine et al., 2021; Bridges et al., 2020; Neath et al., 2011; Pronk et al., 2020; Reimers & Stewart, 2015). Noisy RT measurements may have only a modest effect on the reliability with which experimental effects can be measured (Reimers & Stewart, 2015), as confirmed by online studies that consistently replicated experimental effects found with cognitive tasks (Bazilinskyy & de Winter, 2018; de Leeuw & Motz, 2016; Germine et al., 2012; Hilbig, 2016; Semmelmann et al., 2016; Semmelmann & Weigelt, 2017). However, noise introduced by devices into RT measurements can be an issue when scoring a task based on *absolute RT* (i.e., a score based on an aggregation of RTs across a single condition). For instance, in repeated measure designs, participants using different devices between time points can introduce systematic errors (Reimers & Stewart, 2015). Additionally, device noise may have a relatively strong impact on the measurement of individual differences based on absolute RT (Pronk et al., 2020). In contrast, relative RT (i.e., a score which is based on a difference between aggregated RTs across two or more conditions) is less affected by the aforementioned issues (Bridges et al., 2020; Pronk et al., 2020; Reimers & Stewart, 2015). Hence, for repeated measures designs or individual differences research, we recommend using relative RTs when possible, even though, all things considered, relative RTs may be less reliable than absolute RTs, especially when the RTs of the conditions that are subtracted from each other are correlated (for example, Peter et al., 1993).

### Flanker task design for smartphones

Cognitive tasks requiring speeded responses commonly present participants with a two-alternative forced-choice task, in which a participant provides one of two responses in each trial. However, when a keyboard response device is used, it is easy to increase the number of response options available—for instance, the left and right index fingers for two response options, adding the left and right middle fingers if four response options are required. Smartphone operation is different from keyboard device operation in several ways. Smartphones are generally handheld, have comparatively small screens in a larger variety of aspect ratios, and are operated by pressing touch-sensitive areas on the screen. This limits cognitive tasks in terms of the amount of information that can be presented simultaneously and the number of response options that can be provided conveniently (Passell et al., 2021).

Based on the considerations above, we deem the flanker effect (also known as the incongruency cost effect; Ridderinkhof et al., 2021), as measured via the flanker task, or Eriksen flanker task, suitable for administration on commodity devices: generally speaking, the flanker task does not require particularly brief or precise stimulus timing, and the flanker effect is scored via relative RT by subtracting mean RT in congruent conditions from mean RT in incongruent conditions. Indeed, two studies have replicated the flanker effect with flanker tasks implemented as web applications on desktops and laptops (Crump et al., 2013; Semmelmann & Weigelt, 2017). Extending to smartphones, we expect the flanker task to be suitable as well, as it presents only a limited amount of information on the screen simultaneously (in our paradigm, five stimuli next to each other) and offers two response options. Finally, as it is a relatively popular cognitive task, findings with the flanker task can be informative for the field in general.

### Individual differences in the flanker effect

Concerning individual differences, Hedge et al. (2018) found test-retest intra-class correlation (ICC) coefficients of .40 and .57 in their student sample. In contrast, as reviewed by Ridderinkhof et al. (2021), several studies found test-retest Spearman-Brown adjusted Pearson correlations and ICCs of .77 and higher (Fan et al., 2002; MacLeod et al., 2010; Wöstmann et al., 2013; Zelazo et al., 2014). This difference in reliabilities becomes more striking when taking into account that the number of trials used to score the flanker effect was 240 per condition in Hedge et al. (2018), but up to 96 in the studies reviewed by Ridderinkhof et al. (2021). The samples used in the latter studies were more diverse with regard to age, gender, and education level, lending credence to our hypothesis that a more diverse sample can yield more reliable individual differences in cognitive tasks such as the flanker task.

To examine whether a more diverse sample indeed yields more reliable individual differences in the flanker effect, we compared a relatively diverse sample—recruited from the general population of the United Kingdom—with a student sample, both taking part via their keyboard devices. We hypothesized (H1) that, when the flanker task was administered via keyboard devices, the diverse sample would yield more reliable individual differences than the student sample. To examine whether smartphones measured individual differences in the flanker effect with a reliability that was as high as keyboard devices, we also administered the flanker task to a relatively diverse sample via their smartphones. We hypothesized (H2) that smartphone administration would not yield less reliable measurements of individual differences than keyboard device administration. These hypotheses were preregistered (https://osf.io/zvr6c), with open access data and materials available at https://osf.io/fwx2n.

### Reliability as a function of internal consistency and task length

Our main hypotheses were tested with a design that was similar to Hedge et al. (2018). Specifically, we calculated test-retest ICCs for absolute agreement (Koo & Li, 2016; Mcgraw & Wong, 1996; Shrout & Fleiss, 1979). This reliability estimate takes into account both the strength of the linear relation of the flanker effect between task administrations and the consistency with which the flanker effect ranks participants from low to high. Test and retest were about a week after each other; we assume a flanker effect that is temporally stable within participants (Kopp et al., 2021). Exploratively, we examined a reliability coefficient that makes less strong assumptions about absolute agreement of scores: Pearson correlations (Parsons et al., 2019).

Exploratively, we also examined a method for estimating reliability that makes less strong assumptions about temporal stability, namely split-half reliabilities (Parsons et al., 2019). As splitting methods, we included both Monte Carlo splitting (Williams & Kaufmann, 2012) and permutated splitting (Kopp et al., 2021; Parsons et al., 2019; Williams & Kaufmann, 2012). Note that the explorative analyses in our pre-registration mention a comparison of various other splitting methods. We have restricted ourselves to Monte Carlo and permutated splitting because both have been considered relatively robust (Pronk et al., 2022), so any differences found between them in this study could be informative in recommending splitting methods for future research. Secondly, we examined how split-half and test-retest reliability estimates develop as a function of the number of trials in the flanker task by repeating the analyses with shortened flanker tasks constructed by subsampling trials. This approach is similar to the supplementary analyses of Hedge et al. (2018) for examining whether reliability stabilizes at a certain number of trials. Similar to Williams and Kaufmann (2012), we examined whether reliability estimates of the flanker effect follow the Spearman-Brown prophecy formula for increasing test length.

To the best of our knowledge, this is the first study that systematically compares the reliability of individual differences in a cognitive task effect across samples and device types. The results of this study may yield insight into whether the “reliability paradox” (Hedge et al., 2018) can in part be addressed by employing more diverse samples, accessible through commodity devices (smartphones). Additionally, the results may inform whether tasks meeting the design guidelines we laid out for cognitive tasks on smartphones offer similarly reliable measurements of individual differences as keyboard devices do. Finally, our explorative analyses may give some insight into the temporal stability of the flanker effect and how reliability develops with increasing trial counts.

## Methods

### Participants

For each condition, we aimed to recruit 152 British participants via Prolific (www.prolific.co). For the condition with students, we filtered on participants who were psychology students. For the conditions with diverse samples, we stratified sampling into eight strata of 19 participants. These strata were formed by each combination of three demographic variables: sex (male versus female), age (younger than 45 versus 45 years to 70), and education level (non-academic versus academic). Non-academic was defined as not being a student as well as having either no formal qualifications, or having completed secondary education, high school, or technical/community college as the highest educational level. “Academic” was defined as having completed an undergraduate, graduate, or doctorate degree. For the keyboard conditions, we allowed participants to only take part with a desktop or laptop device, while for the smartphone condition we allowed participants to only take part with a smartphone device.

### Design

The study consisted of three between-subjects conditions: (1) a student sample taking part via a keyboard device (direct replication of setup in Hedge et al., 2018, for a single task) (2) a diverse sample taking part via a keyboard device (diversity of sample extension) (3) a diverse sample taking part via a smartphone (extension of sample and device). Each condition consisted of two sessions, in which an identical flanker task was administered. The sessions were separated in time by one to two weeks.

### Measures

Each trial of the flanker task consisted of one target, which was an arrow pointing left or right; see the task materials repository. The target was flanked by two distractors on the left and two on the right. In the congruent condition, the distractors were arrows pointing in the same direction as the target. In the incongruent condition, they pointed in the opposite direction. All arrows were scaled to 30% of the height of the screen and were black in front of a 50% grey background (of 50% luminance). Each trial started with a fixation cross at the location of the target arrow. The fixation cross had one of 10 randomly selected durations formed by each whole multiple of 50 ms in the range of 500 to 950 ms. Next, the target and distractors were presented, and remained onscreen, until a response was given. On keyboard devices, participants were instructed to press the S key with their left index finger for left-pointing targets and the L key with their right index finger for right-pointing targets. On smartphone devices, participants were instructed to hold their devices with both hands in landscape orientation, pressing a touch-sensitive area in the bottom-left with their left thumb and a touch-sensitive area in the bottom-right with their right thumb, for left- and right-pointing target arrows, respectively.

At the start of the task, it was ensured that the device screen had a sufficiently high aspect ratio (≥ 1.6), barring further participation if this was not the case. Also, it was ensured that the screen was in landscape mode, with participants instructed to turn their device to landscape if this was not the case. Next followed one practice block and two main blocks, with a break after each block. The practice block presented eight trials, balancing target arrow direction and condition. Each of the two main blocks presented each of the 40 combinations of (a) left- or right-pointing target arrow, (b) congruent or incongruent condition, and (c) 10 fixation durations, four times. Hence, a total of 160 congruent and 160 incongruent trials were presented. This number of trials per condition was based on the supplementary analyses of Hedge et al. (2018), which showed reliability stabilizing at about 160 trials per condition. Trials were presented in pseudorandom order.

During each block, feedback was presented when a response was too fast (i.e., a response during the fixation cross) or when a response was incorrect (e.g., target pointed right but the response was left). Feedback was presented for at least one second and required a response to continue to the next trial. In the practice block, feedback was also presented following correct responses; this was aimed to aid participant comprehension of the task instructions.

Also following Hedge et al. (2018), participants were excluded if their accuracy was below 60% in either session (see Results – Participants). RTs below 100 ms and RTs greater than three times each individual’s median absolute deviation (3MAD) were excluded from the analysis. The flanker effect was calculated as the difference in mean RTs for correct responses between the incongruent and congruent task conditions.

The flanker task was implemented in PsychoJS (Bridges et al., 2020), which is the online counterpart of PsychoPy (Peirce, 2007; Peirce et al., 2019; Peirce & MacAskill, 2018). The source code of the task is available at https://osf.io/mhg5e.

### Procedure

The first session consisted of a study briefing and informed consent, after which participants completed the first administration of the flanker task. Participants were requested to complete the second session about a week later. The second session consisted of another administration of the flanker task, followed by a debriefing. At the end of the first session and the beginning of the second session, participants were requested to use the same device for both sessions.

### Data analysis

All data analyses were performed using R version 4.1.1 (R Core Team, 2021). Both hypotheses were tested via one-sided *z*-tests for the difference between Fisher *z*-transformed correlations across independent samples. These correlations were between the magnitude of the flanker effect in the first and second sessions. For both hypotheses, we assumed a medium (*d* = 0.3) effect, but with different levels of type 1 and type 2 errors. For H1 we tested whether the diverse sample on keyboard devices had a higher test-retest reliability than the student sample on keyboard devices with α = 0.05 and β = 0.2, considering a *p*-value < α as evidence for H1. For H2 we tested whether the test-retest reliability of the diverse sample on smartphone devices was as high as the diverse sample on keyboard devices. To this end, we tested whether the diverse sample on keyboard devices had a higher test-retest reliability than the diverse sample on smartphones, with α = 0.2 and β = 0.05, considering a *p*-value ≥ α as evidence for H2. The latter is comparable to a non-inferiority test with the smallest effect size of interest being *d* = 0.3 (Lakens et al., 2018, 2021). For both hypotheses, sufficient power and sensitivity could be obtained with 141 participants, as determined with G*Power 3.1 (Erdfelder et al., 1996). In practice, we oversampled to account for any drop-out and exclusion (see Results for details).

As a primary reliability coefficient, we calculated an ICC for absolute agreement, using two-way mixed effect models (Koo & Li, 2016; Mcgraw & Wong, 1996; Shrout & Fleiss, 1979). As an alternative reliability coefficient that only assesses the linear relation between test and retest scores or parts yielded by a split-half procedure, we calculated Pearson correlation coefficients.

To examine how reliability develops as a function of flanker task length, we constructed flanker datasets with 40, 80, …, 320 trials by subsampling trials (i.e., random sampling without replacement) stratified by arrow direction and congruence. Constructed in this fashion, a 40-trial flanker represents a flanker task of one-eighth of the original length, while a 320-trial flanker is the original dataset in a randomized trial order. Next, we calculated a reliability coefficient via one of three methods: test-retest correlation, permutated split-half, and Monte Carlo split-half. This procedure was replicated 10,000 times, averaging the estimates over replications via a simple mean. Upon suggestion by a reviewer, we also calculated the mean of Fisher *z*-transformed coefficients, followed by back-transforming the mean *z*-transformed value to a correlation coefficient. In line with the findings of Feldt and Charter (2006), the latter approach yielded coefficients that differed from the simple mean approach by 0.01 at most.

We compared the subsampled coefficients with predictions from the Spearman-Brown prophecy formula as follows. First, we assumed 101 full-length reliability coefficients of 0.00, 0.01, …, 1.00. For each of these 101 coefficients, we calculated the reliability coefficients predicted by the Spearman-Brown prophecy formula for a test of ${~}^{1}\!\left/ \!{~}_{8}\right.$, ${~}^{2}\!\left/ \!{~}_{8}\right.$, ${~}^{3}\!\left/ \!{~}_{8}\right.$, …, and full length, yielding 101 reliability curves. Equation 1 shows the Spearman-Brown formula, where ${\rho}_{xx^{\prime }}$ is the reliability of the full-length test, *n* is the length of the shortened test proportional to the full-length test, and ${\rho}_{xx^{\prime}}^{\ast }$ is the predicted reliability of the shortened test. Per group, reliability coefficient (test-retest correlation, permutated split-half, and Monte Carlo split-half), Spearman-Brown curve, and test length, we calculated the squared difference between the subsampled coefficient for test length X and the corresponding Spearman-Brown coefficient for test length X. Per group and reliability coefficient we selected the best-fitting Spearman-Brown curve as the curve whose sum of squared differences over test lengths was the smallest. All split-half analyses were conducted with the splithalfr R package (Pronk, 2021).

1 $${\rho}_{xx^{\prime}}^{\ast }=\frac{{n\rho}_{xx^{\prime }}}{1+\left(n-1\right){\rho}_{xx^{\prime }}}$$

## Results

### Participants

The sampling specification in our first round of data collection did not prevent a subset of participants from taking part in both sample conditions (*n* = 2) or both device conditions (*n* = 34). These participants were excluded; therefore, we recruited new participants to compensate for this data loss. A total of 467 participants started the experiment in a single sample or device condition, of which 466 completed the first flanker and 449 also completed the second flanker. The number of days between the first and second flankers was 6.5 to 7.4 for 75% of participants.

Our pre-registration did not mention dropping any participants with flanker score outliers. However, our preliminary analyses revealed two participants with flanker scores with absolute *z*-values above 15, both of which were in the Diverse Keyboard group. To assess the impact of these two participants on reliability estimates, relative to the exclusion of any other participants, we calculated 10,000 test-retest ICCs of the Diverse Keyboard group, each time excluding two random participants with the restriction that neither was one of the above two outliers. The highest ICC thus obtained was 0.24. Excluding both outliers yielded an ICC of 0.61, giving a reasonable indication that the two identified participants were outliers with high leverage. Hence, we excluded both outliers from succeeding analyses.

All participants met the inclusion criterion of 60% correct in both sessions, with the lowest being 85%. The final sample had 153 participants in the Student Keyboard group, 153 in Diverse Keyboard, and 141 in Diverse Smartphone. Table 1 shows demographics per sample and device condition. As is common for students, the Student Keyboard sample had an age that was lower and less varied than that of the other samples. As tends to be common for psychology students, the Student Keyboard sample had more female participants than male participants, while the other samples had a more balanced sex distribution.

**Table 1:** Demographics per sample and device condition

Demographic	Student Keyboard	Diverse Keyboard	Diverse Smartphone
Mean age (years)	21.6	36.8	37.8
*SD* of age (years)	3.6	15.3	14.1
# Male	27	78	69
# Female	126	75	72
# Low education level		76	71
# High education level		77	70

Additionally, we examined which devices were used to take part by parsing the UserAgent string (Mozilla, 2022). We detected 23 unique combinations of OS and browser, while one UserAgent could not be parsed. In the first session, the five most frequent combinations were: Windows Chrome (*n* = 150), MacOS X Chrome (*n =* 64), Android Chrome (*n =* 62), iOS Safari (*n =* 51), and Mac OS X Safari (*n =* 30). In 20 cases, we detected a different OS or browser being used in the first and second sessions.

### Flanker descriptives

Table 2 shows descriptives of flanker measures. Below, we report on statistically significant differences between congruent/incongruent trials, sessions, and groups, that may aid interpretation of our main hypothesis tests. As dependent variables, we used the flanker effect, mean RTs on congruent and incongruent trials (i.e., flanker score composite measures), trials with incorrect responses, and RT outliers (i.e., excluded trials that might differ between conditions). Count data was tested via Mann-Whitney tests (unpaired) and Wilcoxon tests (paired), mean RTs via t-tests, and variance of RTs via Levene’s tests. The standard flanker effect was found, with scores being greater than zero in all groups and sessions (*p*s < 0.001), indicating slower response times on incongruent as opposed to congruent trials. Additionally, a congruency effect was found on responses that were removed before calculating a flanker effect, namely the number of RTs > 3MAD and incorrect responses (*p*s < 0.001)

**Table 2:** Descriptives of flanker measures per group and session. Con: congruent trials. Inc: incongruent trials. As the median number of RTs < 100 ms was zero across conditions, these are not reported in the table

Measure	Session	Student keyboard		Diverse keyboard		Diverse smartphone	
		Con	Inc	Con	Inc	Con	Inc
Median of % RT > 3MAD	1	3.75	5.00	3.12	5.00	3.12	5.31
	2	3.44	5.00	4.06	5.31	3.44	5.31
Median of % incorrect	1	0.31	1.25	0.31	0.94	0.00	0.62
	2	0.31	1.25	0.31	0.94	0.00	0.62
Mean of mean correct RTs (ms)	1	455	492	498	528	550	592
	2	442	477	485	513	537	578
SD of mean correct RTs (ms)	1	26	24	34	35	26	27
	2	21	20	26	25	31	29
Correlation mean correct RTs congruent and incongruent	1	0.98		0.99		0.97	
	2	0.98		0.99		0.98	
Mean of flanker score (ms)	1	37		30		41	
	2	35		28		42	
*SD* of flanker score (ms)	1	13		18		21	
	2	13		16		19	

Comparing sessions, the mean RTs of correct responses were lower in session 2 than in session 1 for both congruent and incongruent conditions (*p*s ≤ 0.04). None of the other measures showed significant differences between sessions, except for an increase in the number of RTs > 3MAD from session 1 to session 2 in the congruent trials of the Diverse Keyboard group (*p* = 0.005). In none of the groups did flanker scores significantly differ between sessions (*p*s ≥ 0.11). Hence, while participants got faster at the flanker task overall, the flanker effect was relatively constant over sessions.

Finally, we compared the means and variances of flanker scores between groups. For both sessions, mean flanker scores were lower in the Diverse Keyboard group than in the Student Keyboard and Diverse Smartphone groups (*p*s < 0.001). For both sessions, the variance in flanker scores was higher in the Diverse Keyboard group than in the Student Keyboard group (*p*s ≤ 0.006), but they were not significantly different between the Diverse Keyboard and Diverse Smartphone groups (*p*s ≥ 0.12).

### Test-retest reliabilities

Our main hypotheses were tested via *z*-tests on Fisher *z*-transformed ICCs between the flanker scores of sessions 1 and 2. We found no evidence for H1, as the Diverse Keyboard group (*r* = 0.61) did not have a higher test-retest reliability than the Student Keyboard group (*r* = 0.55), *d* = 0.09, *p* = 0.21. We did find evidence for H2, as the Diverse Keyboard group (*r* = 0.61) also did not have a higher test-retest reliability than the Diverse Smartphone group (*r* = 0.63), *d* = −0.03, *p* = 0.62. Pearson correlations were close to ICCs, being at most 0.01 higher, so for the explorative analyses below, we only report ICCs.

### Split-half reliabilities

Exploratively, we analyzed split-half reliability estimates. Overall, these were higher than test-retest reliabilities, with estimates from permutated splits being 0.63, 0.75, and 0.82 for the Student Keyboard, Diverse Keyboard, and Diverse Smartphone groups, respectively. Estimates from Monte Carlo splits were higher still, being 0.71, 0.79, and 0.84, respectively. Testing our main hypotheses on split-half reliability coefficients, we find a higher reliability in the Diverse Keyboard group than in the Student Keyboard group when split permutated (*d* = 0.24, *p* = 0.02), but not when split Monte Carlo (*d* = 0.18, *p* = 0.057). The Diverse Smartphone group did not have a lower reliability than the Diverse Keyboard group, neither split permutated (*d* = −0.17, *p* = 0.92) nor Monte Carlo (*d* = −0.14, *p* = 0.88). Across groups, the distributions of permutated and Monte Carlo split-half estimates were disjoint by at least 27%, suggesting a relatively large effect of splitting method on split-half reliability estimates.

**Fig. 1:**
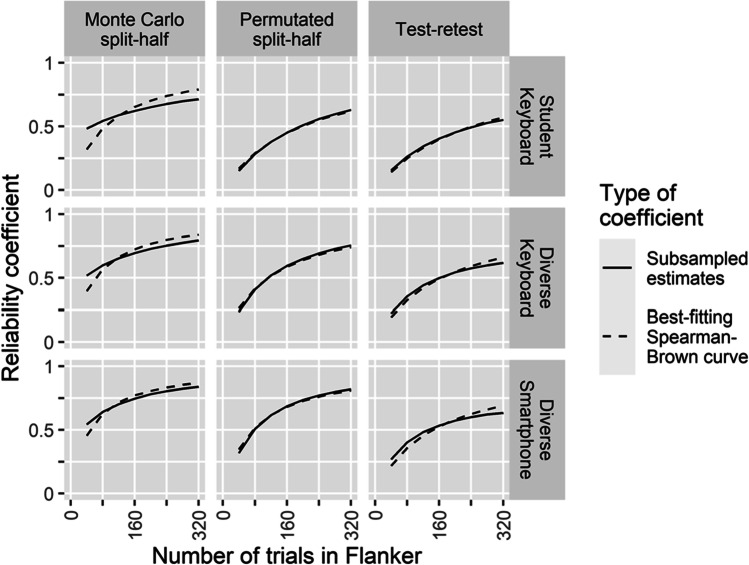
Reliability coefficients as a function of flanker length

Figure 1 shows the reliability estimates obtained via subsampling and best-fitting Spearman-Brown predictions. For permutated and test-retest reliability, Spearman-Brown-predicted curves match the subsampled curves well. Assuming this would also apply to flanker tasks of increased length, we could use the Spearman-Brown formula to predict how long the flanker task would need to be to achieve a test-retest reliability of 0.8. This would require flanker tasks of 1054, 819, and 747 trials, for the Student Keyboard, Diverse Keyboard, and Diverse Smartphone group, respectively. For Monte Carlo split reliability estimates, Spearman-Brown predicted curves did not match the subsampled curves well. Note that, as flanker length approaches zero, one would expect that its reliability estimate would approach zero as well. However, the Monte Carlo estimates stay relatively high. This might indicate that Monte Carlo splits overestimate reliability, especially for relatively short tasks.

## Discussion

Research deployed via the internet and administered via smartphones may have access to more diverse samples than the student samples commonly recruited for lab research (Dufau et al., 2011). Diverse samples could have more variation in their traits. Since reliable measurements of individual differences require variation in the trait measured, more diverse samples could yield more reliable measurements of individual differences. Hence, research deployed via the internet and administered via smartphones could potentially address the issue of cognitive tasks having relatively low reliabilities (coined the “reliability paradox” by Hedge et al., 2018). In the Introduction, we formulated four criteria for determining whether a cognitive task is, in principle, suitable for commodity laptops and smartphones: no very brief or precise stimulus timing, relative response times (RTs), a maximum of two response options, and a small number of graphical stimuli. We identified the flanker task and associated flanker effect as meeting these criteria. Hence, the flanker effect was deemed suitable for testing whether the reliability of individual differences measured with cognitive tasks can be improved by diverse samples and smartphones.

We operationalized reliability as test-retest ICCs for absolute agreement. We hypothesized (H1) that a more diverse sample would yield higher test-retest ICCs for the flanker effect than a student sample. While the diverse sample indeed showed more variation in the flanker effect, we did not confirm that a more diverse sample yields higher test-retest ICCs. Additionally, we hypothesized (H2) that smartphones would not yield lower test-retest ICCs than laptops. In line with H2, smartphones did not show a lower variation in the flanker score nor lower ICCs. Hence, we can confirm that smartphones appeared to be just as suitable for reliably measuring individual differences in the flanker effect as laptops were. Exploratively, we examined an index of reliability that does not assess absolute agreement, but only the linear relationship between test and retest scores (Pearson correlations). Within groups, flanker scores did not differ significantly between test and retest sessions, and Pearson correlations were close to ICCs. Hence, we conclude that differences in reliability between the groups included in this study are mostly reflected in the linear relation between test and retest scores, and not by the absolute agreement between test and retest scores.

At first sight, it might appear counterintuitive that the diverse sample did have a significantly higher variation in the flanker effect than the student sample, but not a significantly higher test-retest reliability. We offer two possible explanations for this result. Firstly, note that descriptively, the test-retest reliabilities were higher in the diverse sample, but not high enough to be statistically significant. Hence, our first explanation is that between-subjects tests of differences between correlations are simply not very sensitive, as also reflected by the relatively large samples we required for detecting a medium effect. Secondly, it may be that the higher variance in the flanker effect as measured by the task did not so much reflect a higher variance in trait, but a higher variance in error.

Test-retest reliabilities require multiple flanker administrations and assume a trait that is relatively stable over time. In contrast, split-half reliabilities can be estimated from a single administration and may be more strongly affected by a participant’s state instead of trait. Exploratively, we analyzed split-half reliability estimates. Overall, split-half reliabilities were slightly higher than test-retest reliabilities. In line with test-retest reliability estimates, we found no differences in split-half reliabilities between devices. In contrast to test-retest reliabilities, we did find a higher split-half reliability in the diverse sample than in the student sample, but only when splitting task data via the permutated method. Taken together, these results could suggest that split-half reliabilities are more sensitive to participant state than test-retest reliabilities, as indicated by the overall higher values of split-half reliability estimates. Split-half methods could yield slightly more sensitive measures of the reliability of a trait than test-retest methods, as we did not confirm our hypothesis on sample differences in test-retest reliabilities, but we did in a split-half reliability.

Finally, we examined how reliability estimates developed as a function of flanker task length by constructing subsampled datasets. For both test-retest and permutated split-half reliabilities, the relation between flanker length and reliability coefficient could be well-modeled by the Spearman-Brown prophecy formula. This quality could be useful for estimating the number of flanker trials required to reach a given reliability level. For instance, in our results, a test-retest reliability of 0.8 would require roughly 750 to 1000 trials. In contrast to the findings of Williams and Kaufmann (2012) and our findings on test-retest and permutated split-half reliabilities, Monte-Carlo split-half reliabilities were not well modeled by the Spearman-Brown prophecy formula. In particular, reliability estimates at low numbers of trials were relatively high, which might indicate that Monte Carlo splitting overestimates reliability in short tasks. Hence, for tasks with relatively low numbers of trials we recommend estimating split-half reliabilities using permutated splitting instead.

Based on modeling work in Pronk et al. (2020), we recommended relative RTs for being more robust against the inaccuracies in RT measurements introduced by commodity laptops, desktops, and smartphones. However, scores based on relative RT may be inherently less reliable than absolute RTs (e.g., Lord et al., 1968). Hence, mental chronometry via web applications might face a challenging impasse: either attempt to use reliable measures based on absolute RT, which could be attenuated by device accuracy, or use less reliable measures based on relative RT that may remain robust against device accuracy. This challenge might be addressed, in part, by applying more sophisticated psychometrics to cognitive tasks. For instance, we found congruence effects not only present in the flanker effect, but also in various other measures traditionally disregarded when scoring a flanker effect, such as RT outliers. Models that take this information into account, such as the diffusion model (Ratcliff, 1978), could offer richer, and perhaps more reliable, measures of mental processes underlying the flanker effect.

A more explicit measurement model might not only be more reliable overall, but also offer a more nuanced interpretation of reliability estimates. For instance, in the context of cognitive tasks, our applications of permutated split-halves and the Spearman-Brown prophecy formula imply a parallel measurement model (Warrens, 2016). Hence, while we interpreted test-retest and split-half reliabilities differently, psychometrically we equated them (Warrens, 2015). Rouder and Haaf (2019) formulated a measurement model for RT data that explicitly distinguishes features of a test and of a construct. This model not only can be useful for obtaining measures of a construct that are relatively independent of task properties, such as the number of trials, but can also offer a more principled interpretation of different approaches to reliability estimation, such as split-half versus test-retest reliability.

In addition to psychometrics, future research could conduct a more comprehensive assessment of the potential of diverse samples and smartphones by including a wider variety of cognitive task paradigms. As a first assessment of this kind, we chose a between-subjects study design since this was relatively robust against learning effects across successive administrations. However, between-subjects comparisons of correlation coefficients require relatively large samples to have sufficient power for detecting moderate effects. Hence, we only examined a single cognitive task, the flanker task. The flanker effect was relatively constant across sessions, suggesting an absence of learning effects. Hence, more comprehensive studies could perhaps improve on power by varying devices and tasks within-subjects. A second avenue could be an examination of procedural differences between the flanker designs of Hedge et al. (2018) and the current study on the one hand, and designs by studies that found higher reliabilities on the other (Fan et al., 2002; MacLeod et al., 2010; Wöstmann et al., 2013; Zelazo et al., 2014). For instance, the majority of these studies feature a cue of varying validity. Such procedural variation might keep the participant more attentive, and so yield higher quality data.

In summary, based on our preregistered hypotheses, we found no evidence that the reliability paradox may be resolved via diverse samples. Hence, students may be just as suitable for individual differences research as a more diverse sample. We found reliability estimates ranging from 0.55 to 0.63 with numbers of trials suitable for online administration (300), from which we carefully draw optimism that a sufficiently reliable flanker task could be feasible. Additionally, for researching individual differences in cognitive tasks online, commodity smartphones may be just as capable as laptops.

We recommend that researchers consider using smartphones for cognitive task research if a paradigm so allows. Given their versatility and ubiquity, smartphones are cost-effective and could be valuable in reaching more diverse or specific samples. Of particular interest could be the increase in scale allowed by online methods. Continuing our careful optimism, we proposed a number of avenues for assessing and improving the reliability of cognitive tasks, as well as increasing the power of designs that compare their reliabilities. A comprehensive examination would require numbers of participants that may be prohibitive for a lab study. However, it may well be feasible online.
